# A Dominance Hierarchy of Auditory Spatial Cues in Barn Owls

**DOI:** 10.1371/journal.pone.0010396

**Published:** 2010-04-28

**Authors:** Ilana B. Witten, Phyllis F. Knudsen, Eric I. Knudsen

**Affiliations:** Neurobiology Department, Stanford University Medical Center, Stanford, California, United States of America; University of Lethbridge, Canada

## Abstract

**Background:**

Barn owls integrate spatial information across frequency channels to localize sounds in space.

**Methodology/Principal Findings:**

We presented barn owls with synchronous sounds that contained different bands of frequencies (3–5 kHz and 7–9 kHz) from different locations in space. When the owls were confronted with the conflicting localization cues from two synchronous sounds of equal level, their orienting responses were dominated by one of the sounds: they oriented toward the location of the low frequency sound when the sources were separated in azimuth; in contrast, they oriented toward the location of the high frequency sound when the sources were separated in elevation. We identified neural correlates of this behavioral effect in the optic tectum (OT, superior colliculus in mammals), which contains a map of auditory space and is involved in generating orienting movements to sounds. We found that low frequency cues dominate the representation of sound azimuth in the OT space map, whereas high frequency cues dominate the representation of sound elevation.

**Conclusions/Significance:**

We argue that the dominance hierarchy of localization cues reflects several factors: 1) the relative amplitude of the sound providing the cue, 2) the resolution with which the auditory system measures the value of a cue, and 3) the spatial ambiguity in interpreting the cue. These same factors may contribute to the relative weighting of sound localization cues in other species, including humans.

## Introduction

The central auditory system infers the location of a sound source in space by evaluating and combining a variety of cues. The dominant localization cues are binaural cues, based on interaural level differences (ILD) and interaural timing differences (ITD), the latter based on measurements of interaural phase differences (IPD) [1]. Because the correspondence between values of ITD and ILD and locations in space varies with the frequency of the sound, the auditory system measures these cues in frequency-specific channels and evaluates them in a frequency-specific manner. The information provided by these cues is combined to create a representation of the most likely location of the acoustic stimulus.

Human psychophysical studies, in which localization cues from different frequencies are put into conflict, demonstrate that these frequency-specific sound localization cues are weighted differentially in determining the location of a sound source. For example, when humans are presented with simultaneous low frequency (500 Hz, 1 kHz or 2 kHz) and high frequency (4 kHz) sounds from different locations, the high frequency sound is grouped perceptually with the low frequency sound (because the sounds are synchronized [2]), and the combined stimulus is lateralized near the position of the low frequency source [3]. In other experiments, low frequency sounds have been shown to alter the lateralization of synchronous high frequency sounds, but not vice versa [4], [5].

These results indicate that the human auditory system follows the rule that low frequency localization cues dominate over high frequency cues when localizing a sound source. The basis for the dominance of low over high frequency cues is thought to be related to the relative spatial resolution provided by each cue. The discriminability index (*d'*), measured psychophysically as the ability to judge whether a sound originates from the left or right of the midline, predicts the relative dominance of localization cues when different frequency components containing conflicting cues are presented simultaneously [5], [6].

We looked for evidence of an analogous dominance hierarchy among sound localization cues in barn owls, and we explored underlying factors that could account for their relative dominance. Owls exploit the same binaural cues for localizing sounds as do humans. However, the human auditory system is only able to measure IPDs for frequencies up to about 1.3 kHz [7], [8] whereas, the barn owl auditory system measures IPD cues up to about 8 kHz [9]. In addition, the barn owl's external ears are asymmetrical which causes the left ear to be more sensitive to high frequency sounds (>3 kHz) from below and the right ear to be more sensitive to high frequency sounds from above [10]. The ear asymmetry causes the ILDs of frequencies above 3 kHz to vary with the elevation of a sound source. Thus, each frequency above 3 kHz provides two binaural cues: an IPD cue that varies with azimuth and an ILD cue that varies primarily with elevation [11]. Hence, for barn owls it is not obvious how sounds will be integrated when low and high frequency cues conflict.

Using an approach similar to that of previous human psychophysical studies [3], [5], we tested the relative dominance of sound localization cues by presenting owls with simultaneous sounds from different locations. The human auditory system uses temporal coincidence as a strong cue to signify that sound components arise from a single object [12]. By presenting owls with synchronous sounds of different frequencies from different locations, we were able to observe the dynamic resolution of contradictory spatial cues as the central auditory system created a neural representation of the inferred location of the stimuli.

## Materials and Methods

### Animals

Adult barn owls were housed in flight aviaries. Birds were cared for in accordance with the US National Institutes of Health Guide for the Care and Use of Laboratory Animals. All procedures were approved by the Stanford University Administrative Panel on Laboratory Animal Care (APLAC).

### Surgeries

Owls were anesthetized with 1% halothane mixed with nitrous oxide and oxygen (45∶55). A small metal fastener was attached to the rear of the skull and recording chambers (1 cm diameter) were implanted over the optic tectum on both sides, based on stereotaxic coordinates, with dental acrylic. A local analgesic (bupivicaine HCl) was administered to all wounds following surgery.

### Behavioral Experiments

Three owls were used for behavioral testing. During training and testing sessions, an owl was placed on a perch in the center of a darkened sound attenuating chamber. The chamber was equipped with remotely-controlled movable speakers mounted on a narrow, horizontal semicircular track of radius 92 cm. The track held two speakers (Audax TM025F1) mounted on a bar that were separated in space by 30°, either in azimuth (horizontal bar) or elevation (vertical bar). Sound bursts consisted of either low (3–5 kHz) or high (7–9 kHz) frequency narrowband noise, 250 ms in duration, with 5 ms rise and fall times. Bandpass filtering was performed digitally using the “ellip” function in Matlab; stopband attenuation was 50 dB. Sound pressure levels (dBA scale), measured at the center of the chamber with the owl removed, were equal (within±1 dB) across frequencies. Head positions were tracked using a head-mounted monitoring device (miniBIRD 500, Ascension Technologies).

During an initial training period, an owl learned to first fixate a zeroing light and then orient its head towards the source of a subsequent sound, which was either the low or high frequency sound. Sound levels were randomly interleaved across a range of 10–60 dB above behavioral threshold measured previously for each owl. The location of the sound source was varied randomly across the frontal ±40° in azimuth and elevation. The owl was rewarded with a piece of meat for orienting toward the sound with a short latency head movement (<500 ms).

During subsequent test sessions, the owl was presented either with one sound alone (as before), or else with two simultaneous sounds (one low and one high frequency narrowband sound) from different locations. When only one sound was presented, the owl was rewarded only when it turned toward the sound source. When simultaneous sounds were presented, the owl was rewarded for any short latency head movement following the onset of the sounds (<500 ms). When both sounds were presented, they were separated either in azimuth or in elevation by 30°. When the sounds were separated in azimuth, the elevation was positioned randomly at either +20°, 0°, or −20°; when the sounds were separated in elevation, the azimuth was positioned randomly at either L20°, 0°, or R20°. Each sound was presented at each relative location with equal probability, and sound levels roved randomly from 10–60 dB above behavioral threshold. Single and paired stimuli were randomly interleaved. The data reported in this paper were collected during these test sessions. We collected 20–30 orientation movements for each stimulus configuration from each owl.

### HRTF measurements

To replicate dichotically the frequency-dependent timing and level content of sounds coming from different spatial positions, HRTFs were recorded from 7 owls, using a method similar to that described by Keller et al. [11]. Briefly, each owl was secured in the center of the sound attenuating chamber using the head fastener, and ketamine (0.1 ml/hr) and vallium (0.025 ml/hr) were administered throughout the session. Probetubes (1.5 cm long) attached to microphones (Knowles FG-23652-P16) were inserted into the ears. The tip of each probetube was placed 1–2 mm from the eardrum and the probetube was attached to the edge of the ear canal with superglue. Broadband sounds (2–11 kHz) from a free-field speaker were presented from positions that spanned the frontal hemisphere in 5° increments. For each speaker position, the signal from each microphone was digitally recorded. The HRTF was calculated for each ear and for each location of the speaker by dividing the Fourier transform of the recorded sound waveform by the Fourier transform of the presented sound waveform. The HRTFs were converted into finite impulse response (FIR) filters, or head-related impulse responses (HRIRs) with a linear-phase FIR filter design using least-squares error minimization [13]. We corrected the HRIRs to account for the filtering properties of the speaker, chamber, probetube, microphone, and earphones (Etymotic ER-1, used for dichotic stimulus presentation) by measuring the appropriate transfer functions (see below), and creating inverse FIRs to cancel out their effects. Corrected HRIRs were then used to filter sound waveforms to simulate free field conditions. The phase angle and amplitude from these HRTFs corresponded to the IPD and ILD as a function of frequency.

### Electrophysiological Experiments

Eleven adult barn owls were used for electrophysiological experiments. During a recording session, an owl was suspended in a prone position with its head stabilized using the mounted fastener. Nitrous oxide and oxygen (45∶55) were administered continuously so that owls remained in a passive state. Sounds bursts, consisting of low (3–5 kHz) and/or high (7–9 kHz) frequency narrowband noise, 50 ms in duration, with 5 ms rise and fall times, were filtered with head-related transfer functions (HRTFs) from a typical barn owl and were presented dichotically through earphones (Etymotic ER-1). HRTFs from different owls are highly consistent across the frontal region of space that was tested [11], [14]. The HRTFs from this owl were chosen because the owl was of average size and its HRTFs closely followed the relationship between ITD and auditory azimuth of the population average. Differences in ILD across the measured HRTFs were on the order of a few decibels. Multiunit and single-unit responses were isolated from the deep layers (layers 11–13) of the OT with insulated tungsten microelectrodes (6–13 MΩ). The identification of the tectal layers was based on distinct unit properties that have been linked to these layers based on electrode track reconstructions [15]. Site selection was based on two properties: 1) robust responses to broadband (2–10 kHz) search stimuli, and 2) neural thresholds to the low and high frequency narrowband stimuli that differed by no more than 20 dB. Spike times were stored using Tucker-Davis (TDT) hardware (RA-16) controlled by customized MATLAB (Mathworks) software. Auditory stimuli were filtered to match those used in the behavioral experiments. Sound levels were set relative to the minimum threshold for the recording site. Each stimulus set was presented 15–25 times in a randomly interleaved manner.

### Data Analysis

The receptive field (RF) center for each site was defined as the position of the weighted average (center of mass) of responses in azimuth and elevation. The RF center measured with the low frequency narrowband sound is referred to as the “low frequency center”, and the RF center measured with the high frequency narrowband sound is referred to as the “high frequency center”.

When deriving population responses to the sounds, before averaging across sites, responses were centered based on the azimuth and elevation of the RF center measured with broadband sounds (2–10 kHz). The low frequency sound was presented 30° to the right of the high frequency sound for 22 sites, and 30° to the left of the high frequency sound for 24 sites. For this second group of sites, the responses were reversed around the center position so that responses across the two groups could be directly compared. For sounds separated in elevation, the low frequency sound was presented 30° above the high frequency sound for 12 sites, and 30° below the high frequency sound for 11 sites. For this second group of sites, for plotting purposes the responses were reversed around the center position so that responses across the two groups could be directly compared. Before averaging across sites, the response strength at each site was normalized to the maximum response, averaged across time, to either sound presented alone.

## Results

### Behavioral responses to two simultaneous sounds separated in space

Owls were trained to respond to free-field low frequency (3–5 kHz) and high frequency (7–9 kHz) narrowband sounds presented alone. Then, they were tested with trials in which low and high frequency sounds were presented simultaneously from different locations, interleaved with trials in which the low and high frequency sounds were presented alone. When paired sounds were presented, the sources were separated in space by 30° either in azimuth or in elevation.

**Figure 1:**
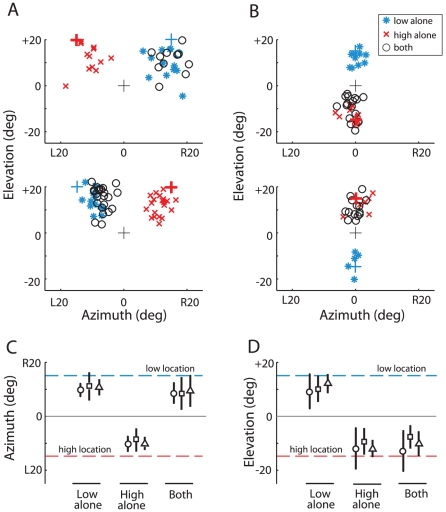
Endpoints of head orienting movements in response to two spatially separated sounds. In **A** and **C**, acoustic stimuli were separated in azimuth; in **B** and **D**, stimuli were separated in elevation. **A**. Data from one owl (Owl L) when sounds were presented either alone or both sounds were presented together, separated in azimuth by 30° and at an elevation of +20°. Top: Azimuth of the high frequency sound was L15°; azimuth of the low frequency sound was R15°. Bottom: Azimuth of the high frequency sound was R15°; azimuth of the low frequency sound was L15°. Blue asterisks represent endpoints of head movements towards the low frequency sound alone; red crosses towards the high frequency sound alone; black circles towards both sounds together. The black cross represents the position of the zeroing visual stimulus; the colored crosses represent the position of the low (blue) and high (red) frequency sound. **B**. Data from Owl L when sounds were presented either alone or both sounds were presented together, separated in elevation by 30° and at an azimuth of 0°. Top: elevation of the high frequency sound was −15°; elevation of the low frequency sound was +15°. Bottom: elevation of the high frequency sound was +15°; elevation of the low frequency sound was −15°. **C**. Average endpoints of the head orienting movements for each of the 3 owls towards each sound alone, and towards both sounds together when the sounds were separated by 30° in azimuth. Sound elevation was either −20°, 0°, or +20° (all conditions randomly interleaved and all data are included in this plot). In the plot, the low frequency sound location is represented by R15° (blue dashed line), and the high frequency location by L15° (red dashed line), although in the experiments both relative positions were tested with equal frequency. Data from the different relative stimulus locations were combined because no statistical difference was observed in responses to sounds either to the right or left of the midline (two-tailed t-test, *p*>.05). Error bars represent STD. 0° corresponds to the position of the visual target for the initial fixation. Each symbol represents a different owl: ○ is Owl B; □ is Owl D; ▵ is Owl L. **D**. Average endpoints for each of the 3 owls towards each sound alone, and towards both sounds together when the sounds were separated by 30° in elevation. Sound azimuth was either −20°, 0°, or +20° (all conditions randomly interleaved). The low frequency sound location is represented by +15° (blue dashed line), and the high frequency location by −15° (red dashed line), although in the experiments both relative positions were tested with equal frequency. Errorbars represent STD. Data from the different relative stimulus locations were combined because no statistical difference was observed in responses to sounds either above or below the visual plane (two-tailed t-test, *p*>.05). Number of head movements reported for each owl: Owl B (200), Owl D (404), Owl L (480). We combined data from the relative orientations because no statistical difference was observed in orienting to sounds to the right and the left of the midline (two-tailed t-test, *p*>.05).

The responses of all three owls were similar. Data from Owl L are shown in Fig. 1A,B. Responses to single sound sources (either low or high frequency) were consistent and stereotyped. The owls responded at short latency (interquartile range = 190–281 ms) with a rapid orientation of the head toward the location of the source. Final orientations consistently undershot the location of the source by errors of 2–5° (mean error for each condition and owl), depending on the source location and the owl.

When the owls were presented with both low and high frequency sounds simultaneously and at equal levels, localization was dominated by the location of the low frequency sound in azimuth and by the location of the high frequency sound in elevation (Fig. 1). The direction of the head turn was highly predictable and the latencies were short (interquartile range = 214–256 ms). When the sources were separated in azimuth, the owls oriented in the direction of the location of the low frequency source, regardless of whether it was to the left or to the right (Fig. 1A, open circles; p<10^−5^ for each owl; 2-tailed t-test; null hypothesis that the mean response was zero). In contrast, when the same stimuli were separated in elevation, the owls oriented in the direction of the high frequency source, regardless of whether it was above or below the horizon (Fig. 1B, open circles; p<10^−5^ for each owl; 2-tailed t-test; null hypothesis that the mean response was zero). Significant differences in orientation latencies were not observed for single and multiple sounds.

**Figure 2:**
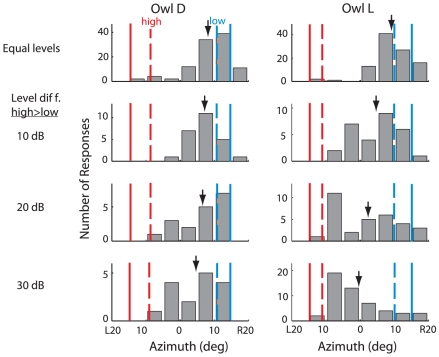
Effect of relative sound level on head orienting movements to simultaneous sounds separated in azimuth. Histograms of endpoints of head orienting movements for Owl D (left column) and Owl L (right column), representing response to all sound elevations. Either the sounds were equal level (1^st^ row), or the level of the high frequency sound was greater than that of the low frequency sound by 10 dB (2^nd^ row), 20 dB (3^rd^ row), or 30 dB (4^th^ row). In the plot, the low frequency sound location is represented by R15° (blue solid line), and the high frequency location is represented by L15° (red solid line), although in the experiments both relative positions were tested equally often. The dashed lines represent the average orientation to each sound presented alone (blue to the low frequency and red to the high frequency). Downward arrows indicate population averages.

The robustness of frequency-dependent localization dominance to changes in the relative levels of the two sounds was tested for azimuthal separations in two owls (Fig. 2). When the low and high frequency sounds were presented at equal levels, the owls oriented toward the location of the low frequency sound: the endpoints of the orienting movements did not differ statistically from those generated in response to the low frequency sound alone (Fig. 2, top histograms; p>0.05, 2-tailed t-test). On interleaved trials, the relative levels of the high and low frequency sounds were altered randomly in 10 dB intervals. As the relative level of the high frequency sound increased, the probability of the owl orientating toward the high frequency sound increased. This effect was more pronounced for Owl L than for Owl D (Fig. 2). Increasing the relative level of the high frequency sound also increased the variance of the responses for both owls. When the level of the high frequency sound had been increased by 15 dB and the level of the low frequency sound decreased by 15 dB (difference = 30 dB; Fig. 2, bottom histograms), Owl D still maintained an orientation bias towards the location of the low frequency sound (*p*<0.001, 2-tailed t-test, null hypothesis that the mean response was zero), whereas Owl L no longer displayed a statistically significant bias in either direction (*p*>.05, 2-tailed t-test).

### Neural responses to sounds separated in azimuth

We recorded neural activity in the OT space map in response to the same low and high frequency narrowband sounds that were used in the behavioral experiments. Auditory neurons in the OT are sharply tuned for space, broadly tuned for frequency, and their spatial tuning is predicted by their tuning to frequency-specific IPDs and ILDs [16], [17].

For these experiments, the owls were sedated and the sounds were presented in virtual space (through earphones; Materials and Methods) to permit rapid interleaving of stimuli from various locations. As in the behavioral experiments, either the low or the high frequency sound was presented alone, or both sounds were presented together with a fixed spatial separation (in virtual space) of 30° in azimuth or elevation. The stimuli were positioned relative to the center of the recording site's RF, and different positions were randomly interleaved. This method of sampling of stimulus space allowed us to infer the distribution of responses across the OT space map to the two stimuli separated in space by 30°.

**Figure 3:**
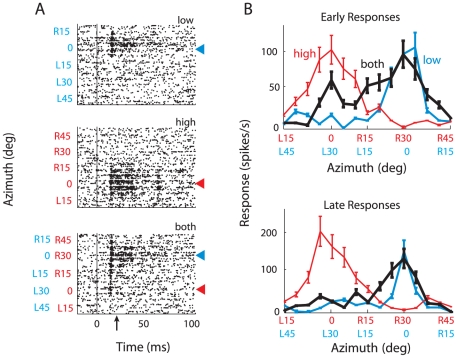
Neural responses at a sample site to individual sounds alone and to paired sounds separated in azimuth. The stimuli were positioned relative to the broadband RF center. A. Raster plots representing responses to the low frequency sound alone (top raster), the high frequency sound alone (middle raster), or both sounds presented together (bottom raster). When both sounds were presented together, the high and low frequency sounds were separated in virtual space by 30°. The position of the low frequency sound is written in blue type on the y-axis, and the position of the high frequency sound is in red type on the y-axis. The values of the low and the high stimuli differed by 30°, reflecting the spatial displacement between the two stimuli. Sound onset = 0 ms; upward arrow indicates 20 ms, the demarcation between early and late responses. Red arrowheads indicate high frequency center; blue arrowheads, low frequency center. **B**. Same data as in A, but displayed as tuning curves representing average response rates plotted separately for the early (top; 0–20 ms) and late (bottom; 20–50 ms) time period. Red curves represent responses to the high frequency sound alone, blue curves to the low frequency sound alone, and black curves to both sounds together. The position of the low frequency sound is written in blue type on the x-axis, and the position of the high frequency sound is in red type on the x-axis.

The responses of the site shown in Fig. 3 were representative of the sites that we sampled (sites that responded well to both the high and the low frequency sounds; Materials and Methods). In all of these experiments, the low and high frequency sounds were presented at sound levels that were equal before being filtered by the transfer functions of the ears, thereby mimicking the stimulus conditions in the behavioral experiment. The site illustrated in Fig. 3 exhibited sharp spatial tuning to either the low or the high frequency narrowband sound when presented alone. The site responded at a latency of 14 ms with a burst of spikes that lasted about 8 ms, followed by a brief decline in firing rate, and then a sustained discharge. Both the phasic and sustained components of the neural response were tuned for the azimuth of the stimulus. The site responded most strongly to either a low frequency sound (Fig. 3A, top raster) or a high frequency sound (Fig. 3A, middle raster) at the RF center (azimuth = 0°). When both sounds were presented together, the site responded at the beginning of the stimulus when either sound was positioned at the RF center (Fig. 3A, bottom raster; 3B, upper plot, black curve). Soon after stimulus onset, however, the pattern of the response changed: the site continued to respond only when the low frequency sound was in the RF (Fig. 3A, bottom, blue arrowhead; 3B, lower plot, black curve). Thus, when the low and high frequency sounds were presented together from different azimuths, the early response to the location of the high frequency sound was suppressed by the presence of the low frequency sound (*p*<10^−3^, two-tailed t-test for most effective high frequency sound position). In contrast, the response to the low frequency sound was not significantly changed by the presence of the high frequency sound (*p*>0.05, two-tailed *t*-test for most effective low frequency sound position).

**Figure 4:**
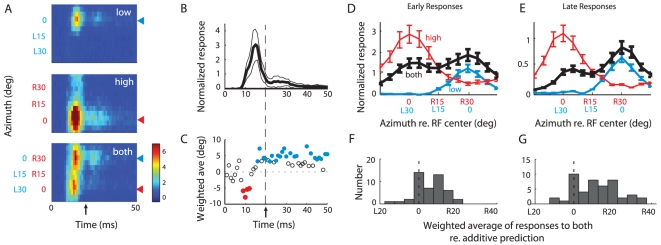
Summary of neural responses to individual sounds alone and paired sounds separated in azimuth. **A**. Population averaged responses as a function of stimulus azimuth and time post-stimulus onset (*n* = 46). Upper panel: responses to the low frequency sound (3–5 kHz).; middle panel: responses to the high frequency sound (7–9 kHz); lower panel: responses to both sounds together. Arrow on time axis demarcates the division between the early and late time period used in panels D and E. **B**. Normalized post-stimulus time histogram, averaged across all stimulus conditions plotted in A. **C**. Weighted average of responses to both sounds together as a function of time (from bottom panel of **A**). Solid circles: statistically shifted from 0 (*p*<.05; bootstrapped *t*-test); open circles: not significantly shifted from 0 (*p*>.05; bootstrapped *t*-test). Leftward shifts: weighted average favors the high frequency location; Rightward shifts: weighted average favors the low frequency location. **D**. Same data as 4A, but displayed as tuning curves for the early time period (0–20 ms). **E**. Same data as 4A, but displayed as tuning curves for the late time period (20–50 ms). **F**. Histogram of the shift of the weighted average of the responses to both sounds together, relative to the weighted average of the additive prediction (the sum of the responses to each sound alone) for each recorded site for the early time period (0–20 ms). Positive values of shift represent a shift towards the low frequency location. **G**. Same as F, but for the late time period (20–50 ms).

**Figure 5:**
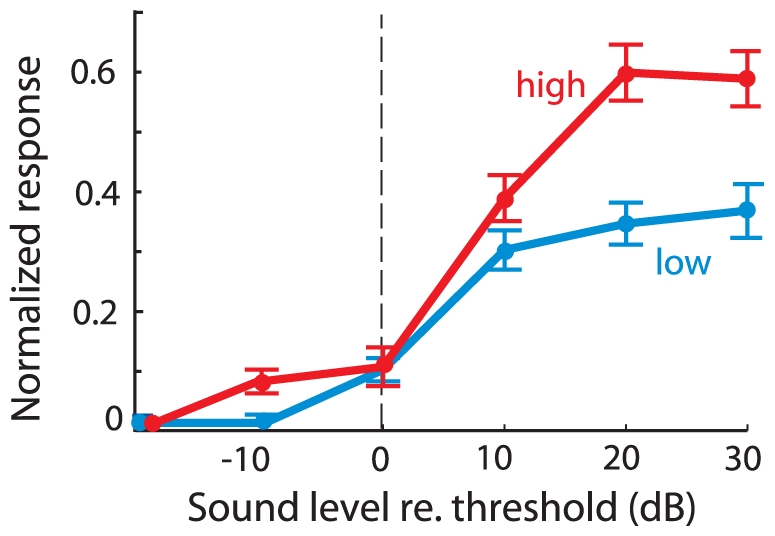
Neural response as a function of average binaural level for the low and high frequency sound. Population averaged neural responses (*n* = 31) to the low (blue) or high (red) frequency sound for the late time period (20–50 ms). Responses at each site were aligned relative to the threshold for each sound and normalized by the maximum response to either sound.

The average response across the population of sampled sites (*n* = 46) is summarized in Fig. 4. Across the population, responses to the low and high frequency sounds presented alone were tuned for source azimuth. In response to the low frequency sound alone, units responded most strongly when the stimulus was centered in the RF (Fig. 4A, top panel) and did not respond when the stimulus was more than 25° to the side of the RF center. Similarly, in response to the high frequency sound alone, the units responded most strongly when the stimulus was centered in the RF (Fig. 4A, middle panel), but they also responded, though at a much lower level, when the source was as much as 45° to the side of the RF center (vertical spread of activity in Fig. 4A, middle panel), reflecting a broader average RF for the high than the low frequency stimulus (mean width at half-max: high = 42°±14°, low = 32°±12°; p<0.01, two-tailed t-test). In addition, the average response to the high frequency sound alone was 48% stronger than that to the low frequency sound alone. This difference in response strength reflected a greater response gain for the high than for the low frequency sound, as verified directly at a subset of sites (Fig. 5).

When the low and high frequency sounds were presented simultaneously from different azimuths, the locations of both the low and the high frequency sounds were represented strongly at sound onset, but soon after, the location of the low frequency sound came to dominate the representation (Fig. 4A, bottom panel). The time-course of the population response (Fig. 4B) was used to define “early” (0–20 ms) and “late” response time periods (20–50 ms). The transition from the representation of both locations to a preferential representation of the location of the low frequency sound occurred during the early phasic response. The dynamics of the transition was analyzed by plotting the weighted average of the population response with 1 ms resolution (Fig. 4C). These data indicate that a rapid shift in the relative representations of the two locations occurred between 12 and 16 ms after sound onset, during which time the representation shifted from a representation centered at the location of the high frequency sound to a representation centered at the location of the low frequency sound.

An analysis of the average activity patterns across the population of recording sites during the early and late phases of the response are shown in Fig. 4D,E. When both sounds were presented simultaneously (Fig. 4D, black curve), the strength of the early response to the two locations was not significantly different (two tailed *t*-test comparing responses to most effective stimulus position, *p* = 0.46), even though the response to the high frequency sound alone (Fig. 4D, red curve) was substantially greater than the response to the low frequency sound alone (Fig. 4D, blue curve; *p*<0.005, two tailed *t*-test comparing responses to most effective stimulus position). The response to the high frequency sound was suppressed by the presence of the low frequency sound (Fig. 4D; red versus black curve; *p*<10^−3^, two-tailed *t*-test for most effective high frequency sound position), whereas the response to the low frequency sound was enhanced by the presence of the high frequency sound (Fig. 4D; blue versus black curve; *p*<0.05, two-tailed *t*-test for most effective low frequency sound position). The opposite effects of the low and high frequency sounds were even more pronounced during the sustained late phase of the response (Fig. 4E, right side). During the late phase, the location of the low frequency sound was more strongly represented than the location of the high frequency sound (Fig. 4E, right side, black curve; two-tailed *t*-test comparing responses to most effective stimulus position, *p*<0.01), even though the response gain to the low frequency sound alone was substantially less than that to the high frequency sound alone during this period (*p*<0.005, two tailed *t*-test comparing responses to most effective stimulus position). Additionally, for both the early and late phases of the population responses, the response to the low frequency sound was enhanced by the added presence of the high frequency sound (Fig. 4D,E, blue versus black curves; *p*<0.05, two-tailed *t*-test for most effective low frequency sound position). In contrast, the response to the high frequency sound was suppressed by the added presence of the low frequency sound (Fig. 4D,E, red versus black curves; *p*<10^−3^, two-tailed *t*-test for most effective high frequency sound position).

To quantify the shift towards the representation of the low frequency sound's location, we compared for each site the center of mass of the responses to both sounds together with the predicted center of mass based on adding the responses to each sound alone (Fig. 4F,G). This analysis demonstrated a shift in the representation of the two sounds that favored the representation of the low frequency location, and the magnitude of the shift was significantly greater during the late phase of the response (early shift: 5±1°; late shift: 11±2°; *p*<10^−3^; two-tailed t-test).

### Neural responses to sounds separated in elevation

**Figure 6:**
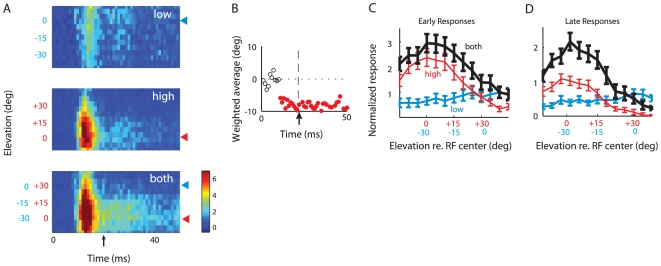
Summary of neural responses to individual sounds alone and to paired sounds separated in elevation. **A**. Population averaged responses as a function of stimulus elevation and time post-stimulus onset to each sound by itself and to both sounds together (*n* = 23). Arrow on time axis demarcates the division between the early and late time period used in panels C and D. **B**. Weighted average of responses to both sounds together as a function of time (from bottom panel of **A**). Solid circles: statistically shifted from 0 toward the high frequency location (*p*<.05; bootstrapped *t*-test); open circles: not significantly shifted from 0 (*p*>.05; bootstrapped *t*-test). **C**. Same data as 6A, but displayed as tuning curves for the early time period (0–20 ms). **D**. Same data as in 6A, but displayed as tuning curves for the late time period (20–50 ms).

OT responses were tuned for stimulus elevation, as well as for azimuth (23 out of 23 sites displayed statistically significant tuning in elevation for the high frequency sound; 18 out of 23 displayed statistically significant tuning for the low frequency; 1-way ANOVA, p<.05). The population average activity revealed that tuning in elevation was much sharper for the high frequency sound alone than for the low frequency sound alone (Fig. 6C,D). When the low and high frequency sounds were presented simultaneously from different elevations, the locations of both sounds were represented in the early response immediately after sound onset, but the location of the high frequency sound dominated the representation in the late response (Fig. 6A,B). Responses to the high frequency sound alone were enhanced by the presence of the low frequency sound (Fig. 6D, left side; red versus black curve, *p*<10^−3^ for two tailed *t*-test comparing late responses to most effective stimulus position). In contrast, responses to the low frequency sound alone were suppressed by the presence of the high frequency sound when the low frequency sound was located near the RF center (Fig. 6D, blue versus black curve, *p*<.01 for two tailed *t*-test comparing late responses for low frequency sound at 5°). Consequently, late responses to both sounds centered on the location of the high frequency sound.

### Effect of relative sound level on the neural representation of location

The effect of changing the relative levels of the low and high frequency sounds was tested at a subset of sites (*n* = 31). To enable a comparison with the behavioral data (Fig. 2), this test was done with the sources separated in azimuth.

**Figure 7:**
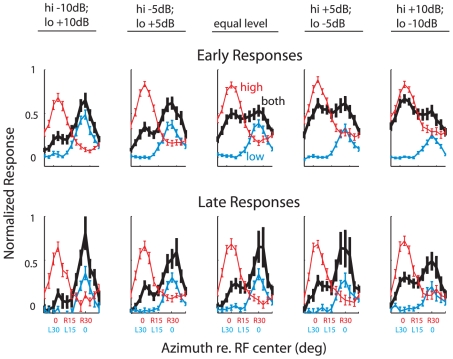
The effect of relative sound level on the representation of sound location. Population averages of the early (top row) and late (bottom row) responses to each sound alone and both sounds together for sounds separated by 30° in azimuth. Red curves: responses to the high frequency sound; blue curves: responses to the low frequency sound; black curves: responses to both sounds together. The relative sound levels for the high and low frequency sounds are indicated above each corresponding row of tuning curves. Error bars indicate standard errors. Neural responses were normalized by the maximum response to either sound alone during the depicted time range, rather than across the entire time range as in Figs. 4,6 (Early: 0–20 ms; Late: 20–50 ms).

Increasing the relative level of a sound increased the relative strength of the representation of its location when both sounds were presented together (Fig. 7). Nevertheless, the location of the low frequency sound continued to be represented differentially strongly, particularly during the late phase of the response, across the range of relative levels tested (Fig. 7, bottom row, black curve).

## Discussion

When low (3–5 kHz) and high (7–9 kHz) frequency sounds of equal level occur simultaneously but originate from different locations, owls behave as though the sounds come from a single location. When the sound sources are separated in azimuth, owls tend to orient to the location of the low frequency sound; when they are separated in elevation, they tend to orient to the location of the high frequency sound. Thus, low frequency sounds dominate localization in azimuth, whereas high frequency sounds dominate localization in elevation.

The pattern of neural activity in the OT space map can explain these remarkable behavioral results. In response to two simultaneous low and high frequency sounds of approximately equal levels, the space map briefly represents the locations of both sounds, then shifts rapidly to a representation that heavily favors the dominant sound (the low frequency sound for sounds separated in azimuth and the high frequency sound for sounds separated in elevation) (Figs. 4,6). Thus, in response to spatially discrepant simultaneous sounds, the auditory space map rapidly and automatically suppresses the spatial representation of the subordinate stimulus and maintains the spatial representation of the dominant stimulus. The frequency-dependence of this effect indicates that the underlying mechanism must operate before the level of the OT, which receives inputs that are already broadly tuned to frequency. In the midbrain pathway that leads to the OT, the likely site is the ICX, where information converges across frequencies to create a map of space [18].

The dominance of the low frequency sound over the high frequency sound, observed for sounds separated in azimuth, was not due to low frequency masking of high frequency information. Low frequency spectral masking refers to the ability of a lower frequency sound to disrupt perception of a higher frequency sound. Our data cannot be explained by spectral masking because the frequency of the stimulus that dominated sound localization depended on whether the stimuli were separated in azimuth or elevation. Low frequency masking would predict low frequency dominance regardless of the direction of the spatial separation.

Most of the data are based on recordings from multiple units. It is likely that some units in the multiunit recordings were responding more to the high frequency stimulus and others to the low frequency stimulus. This likelihood notwithstanding, unit responses to high frequency sounds were suppressed by the presence of a low frequency sound for azimuth separations, and unit responses to low frequency sounds were suppressed by the presence of a high frequency sound for elevation separations. Multiunit recordings increase confidence that this remarkable phenomenon is a general property of the entire population of tectal units.

The stimulus location that owls orient toward behaviorally corresponds to the location represented in the space map >16 ms after sound onset (Figs. 4C and 6B). The transition from an initial representation of both stimuli to a differential representation of just one stimulus progresses during the first 8 ms of the neural response in the OT (Fig. 4C). This implies that the owl's decision of where to orient, if based on OT activity, is determined by the pattern of neural activity more than 16 ms after sound onset, at least when the representation of stimulus location is shifting dynamically (Fig. 4C) due to conflicting spatial cues.

When the level of the subordinate sound is much greater than that of the dominant sound, the owl's orientation responses to the paired stimuli become variable and, in some cases, bimodally distributed (Fig. 2). Neural recordings from the OT space map exhibit a similar pattern. The bimodal distribution of late neural responses when the level of the high frequency sound is much greater than that of the low frequency sound (Fig. 7, lower right), indicates that when the relative level of the subordinate sound increases sufficiently, the representation of the location of the subordinate sound increases.

### A multiplicative rule can explain localization cue dominance

A multiplicative rule for input integration would enhance responses when cues are mutually consistent and would suppress responses when cues are mutually contradictory. A multiplicative rule has been shown previously to operate in the ICX, the processing step before the OT [19], [20]. A multiplicative rule, applied to the neural population data (Figs. 4 and 6), can account qualitatively for the dominance of low frequency cues in azimuth as well as for the dominance of high frequency cues in elevation. In azimuth, the low frequency sound drives no responses when the stimulus is more than 25° to the side of the RF center (Fig. 4D,E; L30°). According to a multiplicative rule, an absence of low frequency input would cancel the effect of the high frequency input, as observed in responses to both sounds together (Fig. 4D,E; black curve). In contrast, the high frequency sound continues to drive responses when the stimulus is located 25° or more to the side of the RF center (Fig. 4D,E; R30°). According to a multiplicative rule, the continued high frequency input would enhance responses to the low frequency sound, as observed. Similarly, the data from the individual site shown in Fig. 3 are consistent with a multiplicative rule operating on sub-threshold inputs [19], [20] that exhibit the same spatial patterns as those of the population data.

A multiplicative rule could also account for the dominance of high frequency cues when sounds are separated in elevation (Fig. 6D). The high frequency stimulus did not drive responses when the sound was located 30° or more above or below the RF center (Fig. 6D, +30°). According to a multiplicative rule, an absence of high frequency input would cancel responses to the low frequency input, as observed. In contrast, the low frequency sound continued to drive responses when the stimulus was more than 30° from the RF center (Fig. 6D, −30°), and this continued drive would enhance responses to the high frequency sound, as observed.

### Relative weighting of low and high frequency cues

The information that is provided by a localization cue depends on its spatial resolution and on the spatial ambiguity in interpreting the cue. The spatial resolution of a cue depends both on the rate at which the cue's value changes with source location and on the ability of the auditory system to discriminate those values. The spatial ambiguity in interpreting cue values arises because most cue values are produced by sounds from many different locations.

**Figure 8:**
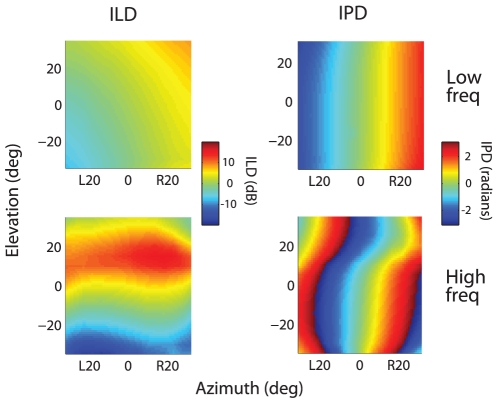
Acoustic spatial cues generated by the low and high frequency sounds. ILD (left) and IPD (right), averaged across 8 owls, for the low frequency (3–5 kHz) and high frequency (7–9 kHz) sounds as a function of the elevation and azimuth of the speaker. IPD and ILD were averaged across the frequency range of each sound.

We propose that, for localizing sounds in elevation, high frequency cues dominate over low frequency cues because of the superior spatial resolution of the high frequency cues and the higher neural gain afforded to high frequency channels. In elevation, the acoustic data show a 3-fold higher rate of change of the ILD cue (dB/deg) for the high frequency sound than for the low frequency sound (Fig. 8, left panels). In addition, in the brainstem nucleus that measures ILD, frequencies above 5 kHz are over-represented and the ILD sensitivity of neurons tuned to frequencies above 5 kHz is greater than that of neurons tuned to lower frequencies [21]. These factors are consistent with the sharper elevational tuning for the high frequency sound relative to the low frequency sound that we observed (Fig. 6). Another factor that favors the representation of the high frequency sound is that, on average, the high frequency sound drives nearly twice as many spikes as does the low frequency sound when each sound is presented alone (Fig. 5). The stronger response to the high frequency inputs may reflect the fact that only the higher frequencies contain high-resolution information about the elevation of the source. Since barn owls are aerial predators, information about the elevation of an auditory stimulus is essential to targeting prey.

In contrast, both high spatial resolution and low spatial ambiguity favor the low frequency cues when localizing in azimuth. The rate of change of the high frequency IPD cue (radians/deg) is twice as large as that for the low frequency cue (Fig. 8, right panels). However, the capacity of the auditory system to encode IPD declines sharply with increasing frequency [22]. We found that the average azimuthal tuning for the high frequency sound was actually less sharp than that for the low frequency sound (width at half-max: high = 42°±14°, low = 32°±12°; Fig. 4D,E), implying that the decline in the auditory system's capacity to encode IPD at high frequencies is more severe than the increase in the rate of change in IPD with sound azimuth. Moreover, the interpretation of the high frequency IPD cue is ambiguous even in frontal space, since equivalent IPD values correspond to different azimuths separated by about 50° (Fig. 8, matching colors). We propose, therefore, that for sound localization in azimuth, low frequency cues dominate over high frequency cues, because of their superior spatial resolution and low spatial ambiguity.

The amplitude of the sound that provides the cue is another factor that influences the contribution of a cue in the determination of stimulus location. As the relative level of a frequency band increases, the neural representation of the sound's location becomes progressively more influenced by the spatial information provided by that frequency band (Fig. 7). This neurophysiological effect could explain the shift in the distribution of behavioral responses that was observed when the amplitude of the subordinate (high frequency) sound was increased to well beyond that of the dominant sound (Fig. 2).

In summary, the data indicate that when inferring the location of a sound source, the auditory system weights the information provided by different cues based on their relative spatial resolution, spatial ambiguity, and the relative amplitude of the sound that provided the cue.

### The representation of multiple sound sources

In this study, we used multiple sound sources to create discrepant spatial cues. Previous studies have used multiple sounds for similar purposes. One group of studies employed the “precedence effect” whereby in response to slightly asynchronous sounds from different locations the auditory system attributes the location from the later sound to the location of the earlier sound. In this case, the auditory system groups the sounds, and differentially weights the spatial cues provided by the earlier sound [23]. Neurophysiological studies have revealed a strong suppression in the representation of the second sound in the auditory space map [24], [25], [26].

In other studies, paired, simultaneous sounds with identical waveforms were presented from different locations to produce a “phantom image” in the auditory space map that was located in between the locations of the two individual sounds [27]. This example of the “stereo effect” is due to acoustic interactions and not to neural processing.

Studies most similar to ours involved presenting owls with simultaneous sounds from different locations, but with overlapping frequency spectra [28]. Unlike in the stereo experiments, the waveform microstructure differed between the two sounds in these experiments. Under these conditions, the owl's auditory system represents the locations of both stimuli. This is because the auditory system evaluates IPD and ILD cues on a millisecond time-scale and, when there are two sources, the relative amplitudes of each frequency component for each source varies on this time-scale. As a result, for any given frequency at any moment in time, one source tends to be represented preferentially and, over time, both sounds are represented. This suggests that the flickering of the represented IPD value and ILD value between two sets of values within frequency channels on a millisecond time-scale is a reliable indicator of the presence of two sources at different locations. In our study, we used non-overlapping frequency bands, thereby eliminating this within-frequency indicator of multiple sources. In the absence of this indicator, spatial information from simultaneous sounds may be integrated according to the rules for cue dominance revealed in this study.

### Comparison with human psychophysics

When humans are presented with simultaneous sounds with non-overlapping spectra from different azimuths, localization is biased towards the location of the low frequency sound, an effect that is reminiscent of the effect we observed in barn owls. In humans, the results indicate that sound localization cues are weighted differentially according to the spatial resolution provided by each cue: the discriminability index (*d'*) of each cue was sufficient to quantitatively predict the rules of integration. In addition, spatial ambiguity may also influence the relative weightings of cues in humans, although the contribution of this factor has not been tested. For humans, spatial ambiguity is not a factor for interpreting IPD because our auditory system does not measure IPDs for frequencies high enough to produce the same IPD value from different azimuths [1], [7]. Spatial ambiguity is a factor, however, for interpreting ILD cues. ILD cues follow complex spatial patterns, and the complexity of the spatial pattern increases with frequency [29]. If spatial ambiguity contributes to the dominance hierarchy of cues for deriving sound source location in humans, as it appears to in owls, then lower frequencies should continue to dominate the localization of higher frequencies even above 4 kHz, for which ILD cues are most important for human localization. A similarity in the rules for the dominance hierarchy of sound localization cues in humans and owls suggests that, in both species, the auditory system infers the location of a sound by weighting differentially the highest resolution and least ambiguous cues.
